# Fully 3D Unrolled Magnetic Resonance Fingerprinting Reconstruction via Staged Pretraining and Implicit Gridding

**DOI:** 10.1002/mrm.70500

**Published:** 2026-07-29

**Authors:** Yonatan Urman, Mark Nishimura, Daniel R. Abraham, Xiaozhi Cao, Kawin Setsompop

**Affiliations:** ^1^ Department of Electrical Engineering Stanford University Stanford California USA; ^2^ Department of Radiology Stanford University Stanford California USA

**Keywords:** deep learning, image reconstruction, magnetic resonance fingerprinting, quantitative MRI, unrolled networks

## Abstract

**Purpose:**

Magnetic Resonance Fingerprinting (MRF) enables rapid quantitative imaging, but high‐resolution 3D reconstructions remain computationally expensive due to the NUFFTs required at every iteration, and the commonly used Locally Low Rank (LLR) regularization becomes ineffective at high acceleration. Learned 3D priors could address these limitations, but training them at scale is challenging due to memory and runtime constraints. This work proposes SPUR‐iG, a fully 3D deep unrolled subspace reconstruction framework that provides fast, high‐quality reconstruction for high‐resolution non‐Cartesian 3D MRF, while keeping training time computationally tractable.

**Methods:**

SPUR‐iG leverages implicit GROG‐based data consistency (DC), which grids non‐Cartesian k‐space using a learned family of kernels, enabling efficient FFT‐based DC with minimal artifacts. To make 3D unrolled training more efficient, we introduce a staged training strategy that keeps computation tractable while progressively improving reconstruction quality. We evaluate the method on a large in vivo dataset, as well as on cross‐vendor out‐of‐distribution data.

**Results:**

At 1 mm isotropic resolution, SPUR‐iG outperforms LLR and a state‐of‐the‐art hybrid 2D‐3D unrolled baseline in subspace coefficient quality and $T_{1}$/$T_{2}$ accuracy. Whole‐brain reconstructions complete in under 15 s, providing up to a 111× speedup for 2‐min scans relative to LLR. Notably, SPUR‐iG reconstructions from 30‐s acquisitions achieve mean $T_{1}$ accuracy that matches or exceeds the mean accuracy of LLR reconstructions from 2‐min acquisitions.

**Conclusion:**

SPUR‐iG introduces a fully 3D unrolled reconstruction framework for MRF that improves both reconstruction speed and accuracy, making high‐resolution accelerated 3D MRF more practical for research and clinical use.

## 1 Introduction

Quantitative MRI (qMRI) measures intrinsic tissue properties such as $T_{1}$ and $T_{2}$, making it less sensitive to acquisition conditions than conventional contrast‐weighted imaging, and thereby enabling consistent comparisons across subjects, time points, and sites [1]. As a result, qMRI has shown promise in studying brain development [2, 3], and tracking subtle neurodegenerative changes [4, 5], as well as in broader clinical and neuroscientific applications [6, 7]. However, qMRI acquisitions are typically slow since they require sampling the temporal signal evolution of each voxel rather than acquiring a single contrast‐weighted snapshot.

Magnetic Resonance Fingerprinting (MRF) [8] is a qMRI technique that encodes tissue parameters through a sequence of varying flip angles (FAs), repetition times (TR), and potentially other acquisition settings across $N_{\mathrm{TR}}$ time points. At each TR, an image is typically acquired using a non‐Cartesian trajectory, producing artifacts that are spatially and temporally incoherent and thus reduce interference with tissue‐specific signal evolutions. After reconstructing the time series of images, voxel‐wise signal evolutions are matched to a dictionary of Bloch‐simulated signals, linking tissue parameters to their expected dynamics and enabling parameter estimation.

While highly efficient to acquire, 3D MRF data is computationally demanding to reconstruct, especially at high resolutions [9, 10]. For example, reconstructing an MRF acquisition of the whole brain with $N_{\mathrm{TR}}=500$ at 1 mm isotropic resolution requires tens of gigabytes to store the time series alone. Moreover, the non‐Cartesian reconstruction necessitates computing non‐uniform FFTs (NUFFTs) at every iteration of the reconstruction algorithm, which can be particularly slow for large 3D problems.

Subspace reconstruction [11, 12, 13] partially addresses this challenge by approximating the signal evolution with a low‐rank basis, thereby reducing dimensionality and implicitly regularizing the reconstruction. The resulting subspace coefficient maps provide a compact, temporally rich representation of the image's signal evolutions across TRs, supporting a wide range of downstream applications, including quantitative $T_{1}$/$T_{2}$ estimation, multi‐compartment modeling [14], and high‐quality clinical contrast synthesis [15, 16].

For highly accelerated MRF, additional regularization beyond the subspace model is required. Locally Low Rank (LLR) [17, 18] regularization is widely used, enforcing low‐rank structure within local patches, but it requires computationally expensive singular value decompositions (SVDs) [19]. At very high acceleration rates, such as acquisitions targeting 1 mm isotropic whole‐brain mapping in under 2 min, LLR alone is insufficient to preserve reconstruction quality.

Broadly, achieving high‐fidelity 3D MRF at these resolutions and acceleration factors is limited by two primary bottlenecks: (1) the reliance on handcrafted priors that lack the expressivity to resolve fine details under high undersampling, and (2) the computationally expensive NUFFTs and regularization operators, which substantially raise reconstruction time and memory usage at high resolutions.

Recent advances address parts of these problems. iGROG [20, 21] enables efficient coil‐based gridding of non‐Cartesian data onto a Cartesian grid using an implicit neural representation (INR) [22] of the gridding kernel, replacing expensive NUFFTs with efficient FFTs at negligible quality loss. This reduces high‐resolution MRF reconstruction times from roughly 30 min to around 4 min.

To overcome limitations of the LLR prior, state‐of‐the‐art unrolled methods such as SOTIP [23] incorporate learned priors via deep learning, yielding substantial gains in image quality. To accelerate reconstruction, SOTIP employs NUFFTs with reduced kernel sizes and oversampling factors, which introduce small artifacts that the denoiser must learn to remove. Additionally, although data consistency (DC) is performed in 3D, denoising is applied slice‐by‐slice in 2D to reduce computational cost.

While such design choices are often necessary for computational efficiency, slice‐wise denoising limits the ability of the model to fully exploit the 3D structure of MRF data, which exhibits volumetric artifacts and spatial correlations. More generally, several works have explored strategies to enable fully 3D learned reconstruction under memory constraints. Memory‐efficient unrolled frameworks based on reversible networks and gradient checkpointing (GC) have demonstrated 3D structural MRI reconstruction with substantially reduced memory footprints, at the expense of increased computation and additional numerical considerations in reversible DC and model layers [24, 25]. Other approaches reduce memory by reformulating the reconstruction. Efficient Cartesian methods exploit separable structure to process smaller reformatted volumes [26], while greedy layer‐wise training schemes perform backpropagation after each unroll iteration [27], reducing memory by roughly a factor equal to the number of unroll steps while keeping the overall computational cost approximately unchanged. However, because intermediate iterates are supervised independently rather than through the final unrolled objective, the optimization target may not fully align with the final reconstruction quality. Spatiotemporal architectures have further extended learned reconstruction to Cartesian dynamic imaging by factoring spatial and temporal processing to improve memory utilization [28].

While these approaches represent important advances, the challenges are further compounded in high‐resolution MRF, where the reconstruction problem is inherently 3D, spatiotemporal, and non‐Cartesian, limiting the direct application of the above methods.

To address these issues, we propose SPUR‐iG (*S*taged *P*retraining for *U*nrolled *R*econstruction with *iG*ROG), a fully 3D deep unrolled reconstruction framework [29, 30, 31, 32]. Our approach combines efficient iGROG‐based DC with a learned 3D prior and a staged training strategy that makes large‐scale unrolled training more efficient. We first pretrain a standalone denoiser with extensive data augmentation, then train with a greedy per‐unroll‐iteration loss to reduce memory demands [27], and finally fine‐tune the full unrolled model using GC [33]. To further improve robustness, the denoiser takes as input both the image and the unroll iteration index, enabling it to amortize performance across varying artifact and noise levels. Evaluated on 45 in vivo MRF acquisitions with retrospective undersampling spanning acquisition times from 2 min down to 30 s, and including an example from an out‐of‐distribution scanner vendor, our framework achieves whole‐brain 1 mm isotropic reconstructions in under 15 s in all cases, while surpassing existing methods in quality.

The contributions of this work are as follows:

1. We present a *fully 3D* unrolled subspace reconstruction method for MRF that improves both the subspace coefficient map quality and quantitative accuracy compared with baseline methods.
2. We achieve 1 mm whole‐brain subspace MRF reconstructions in under 15 s by combining efficient iGROG‐based DC with a model‐based 3D denoiser.
3. We develop a staged training strategy that improves the efficiency of large‐scale 3D unrolled training on moderate hardware and validate it on a large in vivo dataset with diverse acceleration levels, including data from an out‐of‐distribution scanner vendor.

A preliminary version of this work appears in ISMRM 2026 [34].

## 2 Methods

**FIGURE 1:**
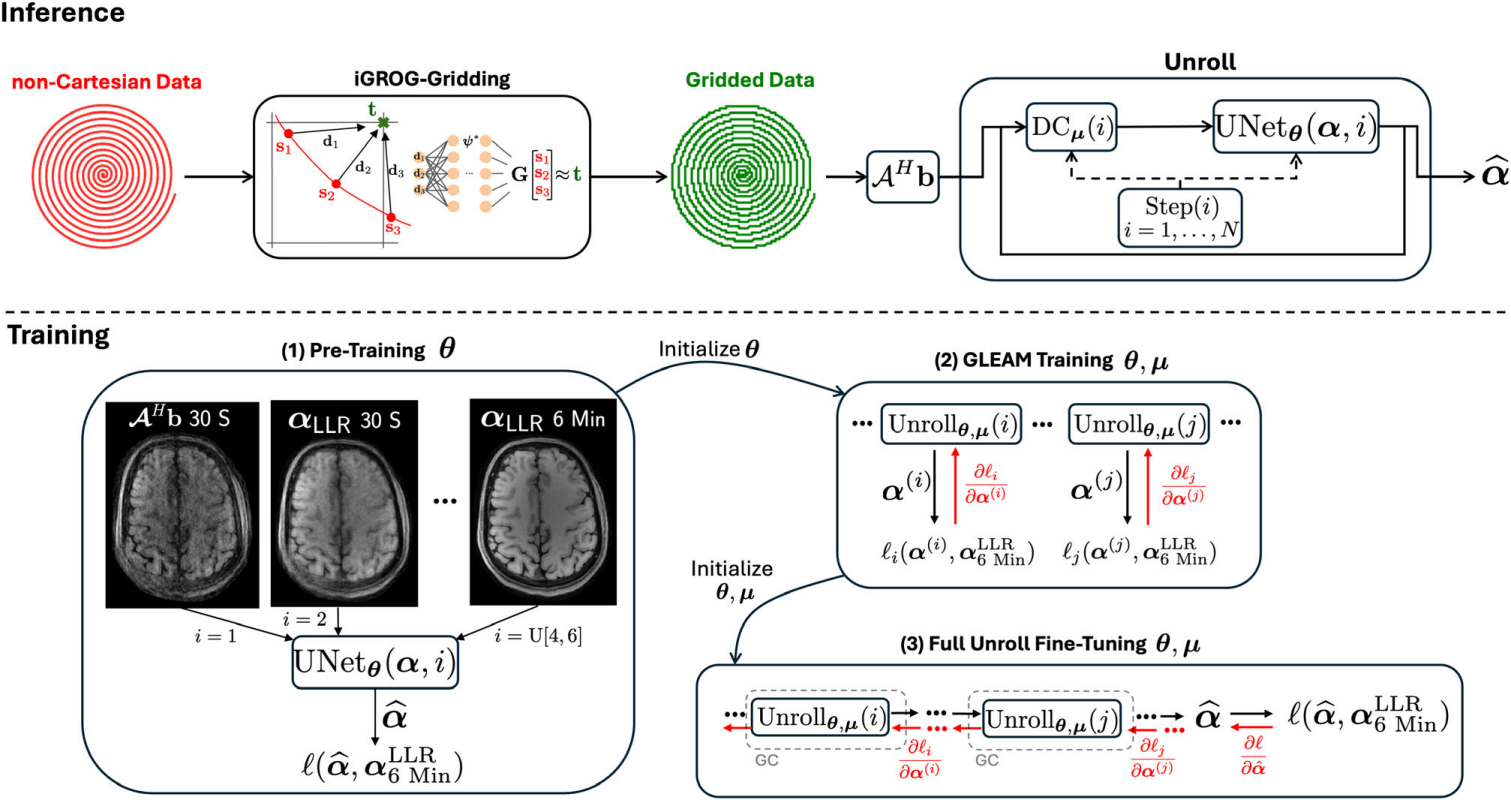
Illustration of inference (top) and training (bottom) pipelines of SPUR‐iG. During inference, iGROG grids the non‐Cartesian data onto a Cartesian grid using an implicit kernel representation learned from the calibration region. The gridded k‐space is then passed to the unrolled reconstruction, initialized with $A^{H}b$. Reconstruction proceeds for N unroll iterations, each alternating between a DC update with a learnable step size $\mu_{i}$ and a step‐conditioned denoiser. Training is performed in three stages: (1) a step‐conditioned UNet is pretrained on inputs with varying artifact levels, using a heuristic mapping between input type and unroll step to promote robustness across noise/artifact conditions; (2) the pretrained UNet is used to initialize a memory‐efficient GLEAM‐style training, where losses are computed and backpropagated after each unroll iteration, detaching subsequent steps; and (3) full unrolled training, initialized from stage (2), fine‐tunes the model under the exact inference setting, with GC across DC and denoising steps to reduce memory consumption.

To enable fast and accurate MRF reconstruction, we build on the standard subspace formulation and address its limitations of suboptimal priors and long reconstruction times. We propose a fully 3D unrolled framework that integrates a learned prior with an efficient DC formulation, along with a staged training strategy that makes large‐scale training feasible within a reasonable compute and time budget. We begin by reviewing MRF acquisition and the subspace model, followed by unrolled reconstruction, and then present the details of our proposed method and evaluation pipeline. An overview of our approach is illustrated in Figure 1.

### 2.1 MRF Subspace Reconstruction

MRF consists of a sequence of $N_{\mathrm{TR}}$ acquisitions with varying excitation parameters (e.g., FAs), producing a time series of images $x\in {\mathbb{C}}^{N_{\mathrm{TR}}\times N_{v}}$, where $N_{v}$ denotes the number of voxels in the volume. Reconstruction typically follows two stages: (1) recovery of the time series $\hat{x}$ from undersampled k‐space data, and (2) voxel‐wise parameter estimation via dictionary matching, where the temporal signal evolution is compared to a precomputed dictionary, and quantitative maps are obtained by selecting the entry with maximum cosine similarity.

Independently reconstructing each of the $N_{\mathrm{TR}}$ images in the time series x is highly ill‐posed due to the high dimensionality and the heavy undersampling used in MRF. This can be mitigated by using temporal redundancy and projecting the data onto a low‐dimensional subspace $\Phi \in {\mathbb{C}}^{N_{\mathrm{TR}}\times k}$ ($k\ll N_{\mathrm{TR}}$) spanned by the top k singular vectors of the MRF dictionary. The time series is then approximated as

(1) x≈Φα

where $\alpha \in {\mathbb{C}}^{k\times N_{v}}$ are the subspace coefficient maps. We define the forward operator acting on α as $A={ℱ}_{u}S\Phi$, with S the coil sensitivities and ${ℱ}_{u}$ the undersampled Fourier operator. Since ${ℱ}_{u}S$ acts on the spatial domain and Φ on the temporal domain, these operators commute, allowing the costly ${ℱ}_{u}S$ to be applied directly in the reduced k‐dimensional space [17].

As acceleration increases, however, the subspace model alone is insufficient, and the reconstruction problem is typically augmented with an LLR prior, leading to

(2) $$\hat{\alpha }=\underset{\alpha }{\arg\;\min}∥A\left(\alpha \right)-b{∥}_{2}^{2}+\lambda \;\mathrm{LLR}\left(\alpha \right),$$

where b are k‐space measurements and LLR(α) enforces low‐rank structure within local spatial patches of the coefficient maps.

### 2.2 Unrolled Reconstruction

Problem (2) is commonly solved using accelerated proximal algorithms such as FISTA [35], which alternate between DC updates and proximal steps. However, at very high acceleration rates, LLR regularization becomes insufficient to constrain the problem, and its proximal operator requires computing SVDs of many local patches at every optimization iteration, making it computationally expensive.

Unrolled reconstruction [29, 30, 31, 32] addresses these issues by replacing the proximal operator with a learned denoiser, yielding the update scheme

(3) $$\alpha_{\frac{1}{2}}^{\left(i+1\right)}=\alpha^{\left(i\right)}-\mu_{i}\;A^{H}\;\left(A\alpha^{\left(i\right)}-b\right),\left(\mathrm{DC}\;\text{step}\right)$$

(4) $$\alpha^{\left(i+1\right)}=f_{\theta }\;\left(\alpha_{\frac{1}{2}}^{\left(i+1\right)}\right),\;\left(\text{learned prior}\right)$$

where $f_{\theta }$ is a learned denoiser and $\mu_{i}$ are learnable step sizes. By unrolling this computation for a fixed number of steps, $f_{\theta }$ can be trained to recover clean images from undersampled inputs, enabling it to learn rich image statistics that surpass hand‐crafted priors.

### 2.3 SPUR‐iG Reconstruction

In large‐scale 3D non‐Cartesian settings, unrolled methods face two key challenges: (1) DC updates remain slow, as evaluating $A^{H}A\left(\cdot \right)$ in (3) requires NUFFTs at every unroll iteration, and (2) training fully 3D unrolled networks is memory‐intensive and slow, since the entire unrolled computation graph must be stored for backpropagation. We address these challenges with an efficient DC implementation and a scalable, progressive training framework.

#### 2.3.1 Efficient DC via iGROG

The main cost in (3) arises from evaluating $A^{H}A\left(\cdot \right)$, which relies on NUFFTs. To accelerate this step, we use iGROG, which learns an INR of the gridding kernel across multi‐channel coils. Using a fully sampled center‐of‐k‐space calibration region (the same data used for coil sensitivity estimation), the INR is trained to map n offset vectors $d_{1},\ldots ,d_{n}$, corresponding to displacements between non‐Cartesian samples and a target Cartesian grid point, to a kernel $G_{\psi^{*}}\left(d_{1},\ldots ,d_{n}\right)$. For each target grid location, nearby source samples are selected, the kernel is evaluated at their relative displacements, and the resulting kernel weights are used to compute the target k‐space value. This enables GRAPPA‐like [36] interpolation and, through the use of an INR, generalizes across the diverse orientations encountered in the trajectory. This allows us to first grid the non‐Cartesian data onto a Cartesian grid, after which NUFFTs can be replaced by efficient FFTs. The upper left part of Figure 1 illustrates this process.

The approach provides flexibility to trade off accuracy and noise amplification against speed. By using an oversampled grid, interpolation quality improves (since the interpolation distances are smaller), but reconstruction time increases. In our experiments, we used a ×1.5 oversampling ratio with 5 k‐space input points per output, achieving high‐quality reconstructions while retaining substantial speed‐ups. Across all acquisitions considered in this work, INR training completed in under 25 s and can be performed during MRF acquisition immediately after calibration data are acquired. Once trained, gridding of the dataset is likewise efficient, completing in under 3 s and yielding data on a Cartesian grid for subsequent FFT‐based DC updates.

#### 2.3.2 Efficient Training of a 3D Unrolled Network

For $f_{\theta }$ in (4), we use a fully 3D UNet [37, 38, 39, 40], which enables the model to capture volumetric context and learn an effective unrolled denoising strategy. To improve data efficiency, weights are shared across unroll steps, and FiLM‐based [41] conditioning is introduced on the unroll step index i, that is, $f_{\theta }\left(\alpha ,i\right)$. In an unrolled reconstruction, the denoiser is applied at each step of the iterative process. The input to the denoiser thus changes substantially across unroll steps: early steps contain heavy aliasing and noise, while later steps, closer to convergence, are substantially cleaner. By conditioning on the unroll step index, the network adapts its denoising behavior to the expected artifact level at each step, rather than having to infer it from the input alone. This is analogous to time conditioning in diffusion models [42, 43].

To enable training of a 3D unrolled network in the challenging MRF reconstruction setting, we adopt a progressive three‐stage strategy, illustrated in the lower part of Figure 1. Throughout all stages, the training target is the ground truth subspace coefficient maps obtained from an R=1 acquisition. Each stage is initialized from the converged weights of the preceding stage, enabling progressive refinement of the model. This strategy consists of the following stages:

1. *Denoiser pretraining:* We begin by pretraining a standalone, unroll step‐conditioned 3D UNet on a diverse set of inputs, where the model is trained to recover clean images from noisy, undersampled inputs. To obtain a robust initialization for subsequent stages, we expose the network to a wide range of artifact and noise levels at its input, including zero‐filled ($A^{H}b$) and LLR‐based reconstructions from scans with different acquisition durations (30 s, 1 min, 2 min, and 6 min, denoted $\alpha_{\mathrm{LLR}}^{30s},\ldots ,\alpha_{\mathrm{LLR}}^{6\min}$). Since the UNet is step‐conditioned, but this stage does not involve actual unrolling and therefore has no natural step index, we assign heuristic pseudo‐step indices to each input type. Specifically, $A^{H}b$ is paired with index 1, corresponding to the initialization of unrolled reconstruction, while reconstructions with increasing acquisition durations are mapped to higher indices (e.g., $\alpha_{\mathrm{LLR}}^{30s}\to 2$). Because this stage does not involve expensive unrolling or DC updates, we can efficiently apply extensive data augmentation, including random rotations, translations, and scaling. At the end of this stage, the network provides a well‐initialized denoising prior that generalizes across a range of artifact levels and incorporates a notion of the unroll step index.
2. *Greedy unrolled training (GLEAM* [27]*):* The goal of this stage is to resolve the input distribution mismatch of $f_{\theta }$. In Stage 1, the denoiser is trained on proxy inputs that provide a good initialization but do not reflect the actual inputs encountered during unrolled reconstruction. Here, the denoiser is trained within the unrolled pipeline, where its inputs are the intermediate outputs of preceding unroll steps, adapting it to the true input distribution.
  To keep training fast, we use a greedy scheme: at each training iteration the full unrolled reconstruction is executed, but the loss after each unroll step is backpropagated independently, and the computation graph is then detached before proceeding to the next step. This allows training on actual mid‐unroll inputs while avoiding the need to store the entire computation graph, which would either exceed typical GPU capacity or, with GC, incur substantial runtime overhead. Combined with the informed initialization from pretraining, this achieves fast convergence. Note, however, that the greedy objective is a proxy: optimizing each intermediate output independently does not account for how earlier steps affect downstream ones, so the resulting solution may not minimize the output reconstruction error. To avoid over‐committing to the GLEAM objective, which could make subsequent fine‐tuning difficult, we apply early stopping before full convergence (details in Section S1.3).
3. *Final fine‐tuning:* We fine‐tune the model using full end‐to‐end unrolled training, initialized from the GLEAM model. In contrast to GLEAM, where gradients are detached after each unroll step and each step is optimized independently of downstream steps, this stage backpropagates gradients through the entire unrolled pipeline, directly optimizing the denoiser with respect to the final reconstruction output. To fit the full computation graph in memory, we employ GC across both DC and denoising steps, which significantly increases the computation time per training iteration. The informed initialization is therefore critical, as it provides a strong starting point that enables convergence in substantially less training time despite the high per‐iteration cost.

Collectively, these stages enable efficient large‐scale training by progressively mitigating the limitations of each preceding stage, substantially reducing the training time relative to training a fully 3D unrolled model from scratch. This process ultimately yields a high‐quality, fully 3D unrolled reconstruction model. We refer to the resulting framework as *SPUR‐iG*, introduced in Section 1. Code will be made publicly available upon publication.

### 2.4 Dataset

We used a FISP‐MRF sequence [44] with a 3D Spiral Projection Imaging (SPI) acquisition [45], beginning with an inversion pulse (TI=20 ms) followed by $N_{\mathrm{TR}}=500$ TRs (TE=1.7 ms, TR=12.5 ms). Each block of 500 TRs was extended by 40 additional TRs to acquire a low‐resolution (4 mm isotropic) navigator for motion estimation [46]. The combined 540 TRs form an acquisition group with a total duration of 7.9 s, including a 1.2 s resting delay between groups to enable $M_{z}$ recovery. Multiple groups are acquired using different spiral projection interleaves and rotations to build up sufficient k‐space encoding per TR position, with sampling patterns optimized for k‐space coverage and incoherent aliasing using Tiny Golden‐Angle Shuffling (TGAS) [9]. Each spiral readout is 6.7 ms long and follows a variable‐density design with in‐plane acceleration factors of 16 (center) and 32 (outer k‐space). In TGAS, each spiral arm is rotated across acquisition groups and TRs along two axes, with in‐plane rotation angle $\Theta_{z}\left(i\right)=22.5{}^{\circ}\times i$ and through‐plane rotation angle $\Theta_{x}\left(i,j\right)=23.63{}^{\circ}\times \left(i+j\right)$ where i is the group index and j is the TR index within a group. The acquisition resolution was 1 mm isotropic with a 220×220×220mm field of view. Two tailored dummy‐scan groups (1080 TRs total, 16 s) were acquired at the start for $B_{1}$ mapping (using the Bloch–Siegert method), with the final 120 TRs using the same constant FA as the regular acquisition groups to ensure steady state is reached before MRF data collection begins, following the design in [47].

Following the convention from TGAS [9], we define an R=1 acquisition as 50 groups (including the two dummy groups), corresponding to a total scan time of 6 min 38 s. We note that R=1 does not imply full Nyquist sampling, but has been shown to produce high‐quality reconstructions suitable for use as ground truth [9]. We considered undersampling factors of R=3,6, and 12, yielding scan times of 2 min 23 s, 1 min 19 s, and 47 s, respectively. Retrospective undersampling was performed in an interleaved fashion along the acquisition‐group dimension, following [9], which optimizes k‐space coverage and spatio‐temporal incoherent aliasing. Rather than selecting a subset of the *R* = 1 groups, each undersampled acquisition group is constructed from rotated spiral interleaves drawn from several different *R* = 1 groups. For reporting, we use the raw MRF acquisition time (excluding the fixed dummy‐group overhead), which we round and denote as 6 min, 2 min, 1 min, and 30 s for convenience. The iGROG INR training and coil sensitivity estimation both require a fully sampled center‐of‐k‐space region; we used a region of size $24^{3}$, extracted from the dummy‐scan groups. Alternatively, a short dedicated calibration scan can be acquired separately. All scans were coil‐compressed to 10 virtual coils, and coil sensitivity maps were estimated using ESPIRiT [48].

The dataset comprised 44 in vivo subjects scanned on a 3 T GE SIGNA Premier system with a 48‐channel head receiver coil, with 36 used for training, 4 for validation, and 4 for testing. To assess cross‐vendor generalization, we additionally evaluated our method on a subject scanned on a 3 T Siemens MAGNETOM Vida system with a 20‐channel head coil. Both systems used the same MRF sequence with identical trajectory design, timing parameters, and RF pulses (2.4 ms hard pulses). The primary hardware differences are the coil array, field strength, and ADC dwell time (4 μs for GE vs. 5 μs for Siemens), which results in slightly different k‐space sample locations along each spiral readout. For all subjects, motion was corrected in k‐space using navigator data [46]. The R=1 acquisition was reconstructed using subspace reconstruction with light LLR regularization via FISTA and served as the training target. Quantitative maps were estimated using a $B_{1}$‐corrected dictionary to mitigate systematic $T_{2}$ bias [49].

To improve numerical stability in SPUR‐iG, which performs joint denoising across subspace coefficient maps, we do not directly use the raw SVD basis Φ derived from the MRF signal dictionary, as later components contain substantially less energy. Instead, we apply a unitary transformation to Φ that approximately equalizes the energy across subspace channels (details in Section S2). This transformation is invertible and leaves the reconstruction solution unchanged, while redistributing energy more evenly across coefficients and thereby stabilizing the training of the learned denoisers. For a fair comparison, the balanced basis was used for all baseline methods.

Finally, we synthesized MPRAGE images from the R=1 quantitative maps using Bloch simulation and used them to obtain segmentation masks with FreeSurfer [50]. These masks were then used to apply region‐specific loss weighting during model training (additional details in Section S1.2).

### 2.5 Evaluation

We evaluated our approach on the held‐out test set using retrospectively undersampled acquisitions of 2 min, 1 min, and 30 s. Evaluation considered both the reconstruction quality of the subspace coefficient maps and the resulting quantitative $T_{1}$/$T_{2}$ maps, obtained by voxel‐wise dictionary matching with $B_{1}$‐corrected dictionaries. In addition, we performed ablation studies to analyze the contribution of different components of our framework.

We benchmarked against two baselines:

1. *LLR:* Subspace reconstruction with FISTA and LLR regularization. The regularization weight was tuned on the validation set for each scan duration to minimize the sum of the mean relative $T_{1}$ and $T_{2}$ errors within WM and GM. The number of iterations was fixed at 40, ensuring convergence.
2. *Hybrid 2D/3D:* Inspired by the state‐of‐the‐art SOTIP [23], this baseline alternates 3D DC updates with axial slice‐wise 2D denoising. For a fair comparison, we train this model using the same progressive procedure. Unlike SPUR‐iG, the denoiser here is a 2D UNet applied independently to each slice at every unroll iteration, with the 3D volume subsequently formed by stacking the denoised slices. Since GPU memory is typically the bottleneck in training 3D unrolled models, we increased the number of channels in the 2D UNet so that both models saturate the memory of a single NVIDIA A6000 GPU (48 GB) during final fine‐tuning, giving all models maximum expressivity under the same hardware constraint. We verified that this increase does not cause overfitting and has negligible impact on reconstruction time.

Separate models were trained for each scan duration. For the unrolled models, we used six unroll iterations, selected based on ablation studies (Section 3.3). Additional implementation details are provided in Section S1.

## 3 Results

### 3.1 Coefficient Reconstruction

Table 1 shows the PSNR and SSIM for the five balanced subspace coefficients across scan durations and reconstruction methods. SPUR‐iG consistently achieves higher PSNR and SSIM compared to both LLR and the hybrid 2D/3D method, with performance gaps widening as scan duration decreases. Statistical testing confirms that our method outperforms both baselines with p<0.001 across all metrics and subspace dimensions.

**TABLE 1:** PSNR (left) and SSIM (right) across the five balanced subspace coefficients (c1–c5).

		PSNR (dB) ↑					SSIM ↑				
	Method	$c_{1}$	$c_{2}$	$c_{3}$	$c_{4}$	$c_{5}$	$c_{1}$	$c_{2}$	$c_{3}$	$c_{4}$	$c_{5}$
2 min	SPUR‐iG (Ours)	**33.9** ± **2.1**	**33.4** ± **2.4**	**33.2** ± **2.3**	**33.0** ± **2.3**	**33.3** ± **2.3**	**0.94** ± **0.02**	**0.88** ± **0.03**	**0.89** ± **0.02**	**0.89** ± **0.03**	**0.89** ± **0.03**
	Hybrid 2D/3D	32.5 ± 1.9	32.6 ± 2.3	31.9 ± 2.1	32.1 ± 2.1	32.1 ± 2.3	0.93 ± 0.03	0.86 ± 0.03	0.87 ± 0.03	0.87 ± 0.03	0.89 ± 0.03
	LLR	31.5 ± 1.6	30.9 ± 2.0	31.5 ± 1.7	31.3 ± 1.8	31.0 ± 1.9	0.90 ± 0.03	0.84 ± 0.04	0.85 ± 0.03	0.85 ± 0.03	0.85 ± 0.03
1 min	SPUR‐iG (Ours)	**31.2** ± **2.2**	**31.2** ± **2.7**	**31.0** ± **2.3**	**30.8** ± **2.4**	**31.3** ± **2.3**	**0.90** ± **0.04**	**0.82** ± **0.04**	**0.84** ± **0.03**	**0.84** ± **0.04**	**0.83** ± **0.04**
	Hybrid 2D/3D	29.8 ± 2.4	30.2 ± 2.4	29.6 ± 2.4	29.3 ± 2.5	29.0 ± 2.8	0.87 ± 0.05	0.80 ± 0.04	0.81 ± 0.04	0.81 ± 0.04	0.80 ± 0.04
	LLR	28.3 ± 2.0	28.9 ± 2.1	28.2 ± 2.0	27.7 ± 2.1	27.1 ± 2.0	0.80 ± 0.06	0.72 ± 0.05	0.74 ± 0.04	0.75 ± 0.04	0.72 ± 0.05
30 s	SPUR‐iG (Ours)	**28.8** ± **2.5**	**29.7** ± **2.8**	**28.8** ± **2.7**	**28.6** ± **2.6**	**29.4** ± **2.5**	**0.86** ± **0.06**	**0.78** ± **0.06**	**0.80** ± **0.04**	**0.79** ± **0.05**	**0.78** ± **0.05**
	Hybrid 2D/3D	27.5 ± 2.8	27.5 ± 2.8	26.9 ± 2.8	27.1 ± 3.1	27.3 ± 2.6	0.82 ± 0.06	0.75 ± 0.06	0.76 ± 0.05	0.76 ± 0.05	0.74 ± 0.06
	LLR	24.5 ± 2.0	24.7 ± 2.5	23.5 ± 2.0	24.5 ± 2.3	25.1 ± 2.1	0.72 ± 0.07	0.63 ± 0.06	0.67 ± 0.06	0.65 ± 0.05	0.65 ± 0.06

*Note:* Results are shown for three scan durations: 2 min (top), 1 min (middle), and 30 s (bottom). The reference for all metrics is the *R* = 1 reconstruction. Metrics are computed on axial brain slices (excluding non‐brain slices) and averaged across slices from all test subjects. Values are reported as mean ± standard deviation across slices. Bold entries indicate values that are statistically significantly larger than those of the other methods.

**FIGURE 2:**
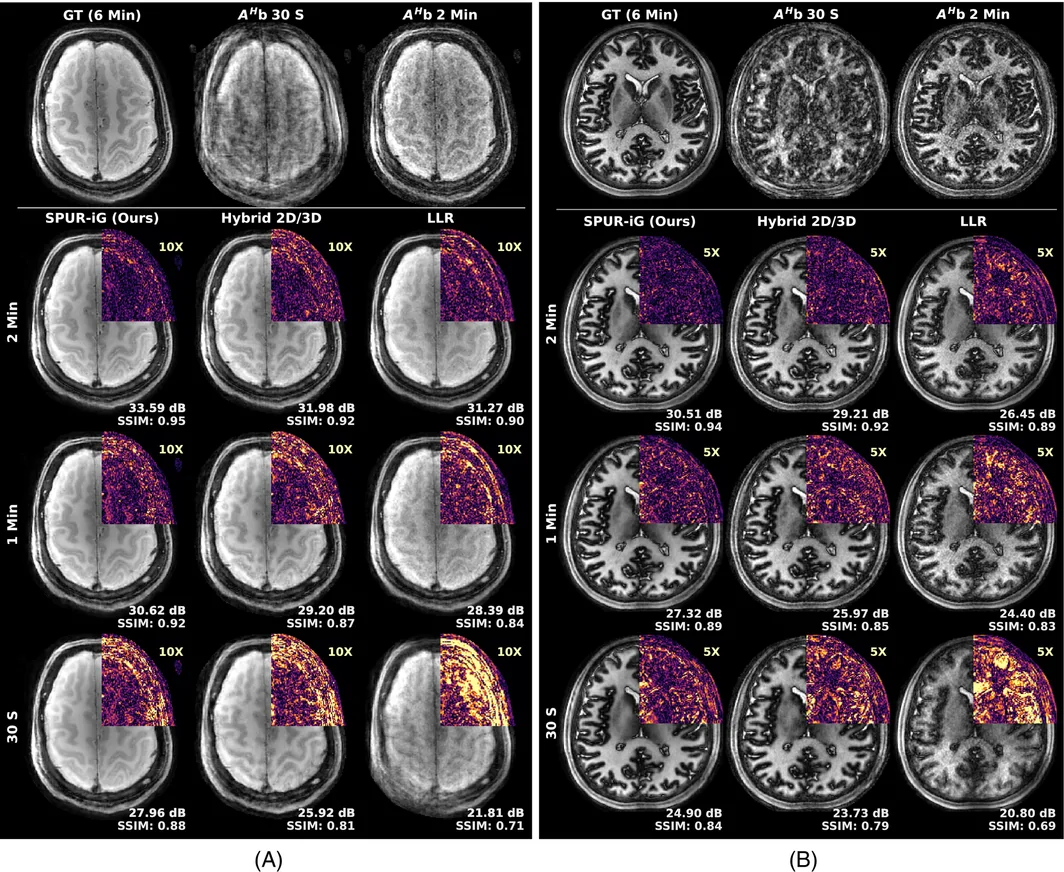
Example reconstructions of the first balanced subspace coefficient (a) and third unbalanced coefficient (b) for two test subjects. The top row shows the 6 min reference (left) and $A^{H}b$ for the 30 s and 2 min acquisitions (middle and right) as initialization references. Subsequent rows correspond to reconstructions at 2 min, 1 min, and 30 s, with columns comparing our method, the hybrid 2D/3D variant and LLR. PSNR and SSIM are reported in the bottom right of each image. Error maps of the top‐right quadrant (magnified ×10 or ×5) are overlaid to illustrate error levels.

Representative qualitative results are shown in Figure 2 for the first balanced and third unbalanced subspace coefficients. These were chosen to display since balanced coefficients generally display flatter contrast, whereas unbalanced coefficients, particularly the third, exhibit higher tissue contrast and reveal finer anatomical details. As scan time decreases, all methods exhibit increased noise, but our approach shows the smallest degradation. For the 30 s scans, both LLR and the hybrid 2D/3D variant produce noisy and over‐smoothed reconstructions, whereas our method better preserves fine structure and anatomical details.

### 3.2 Quantitative Reconstruction

Table 2 shows the average relative quantitative $T_{1}$ and $T_{2}$ errors inside the brain (excluding CSF), defined as $100\cdot |T^{\mathrm{GT}}-\hat{T}|/T^{\mathrm{GT}}$ for $T\in \left\{T_{1},T_{2}\right\}$, where $\hat{T}$ denotes the estimated parameter, along with SSIM values computed on the quantitative maps. Across all scan durations, our method achieves lower errors than both baselines. As expected, accuracy decreases with shorter acquisitions, but degradation is less pronounced for our method. Notably, for $T_{1}$, our reconstructions of the 30 s scans achieved lower average error than LLR reconstructions of 2 min scans. For $T_{2}$, our approach also provides consistent improvements. Statistical testing confirms that our method outperforms both baselines with p<0.001 for $T_{1}$ and $T_{2}$ across all scan durations.

**TABLE 2:** Relative quantitative errors (%) (left) and SSIM (right) of $T_{1}$ and $T_{2}$ maps across methods and scan durations: 2 min (top), 1 min (middle), and 30 s (bottom).

		Relative error (%) ↓		SSIM ↑	
	Method	$T_{1}$	$T_{2}$	$T_{1}$	$T_{2}$
2 min	SPUR‐iG (Ours)	**3.5** ± **2.6**	**4.2** ± **3.2**	**0.915** ± **0.02**	**0.881** ± **0.01**
	Hybrid 2D/3D	4.2 ± 3.1	4.7 ± 3.6	0.904 ± 0.04	0.870 ± 0.04
	LLR	7.2 ± 5	6.4 ± 4.5	0.860 ± 0.09	0.835 ± 0.06
1 min	SPUR‐iG (Ours)	**4.9** ± **3.6**	**5.8** ± **4.3**	**0.864** ± **0.04**	**0.817** ± **0.04**
	Hybrid 2D/3D	5.8 ± 4.2	6.9 ± 5.1	0.836 ± 0.06	0.782 ± 0.09
	LLR	8.2 ± 5.6	8.3 ± 5.9	0.801 ± 0.06	0.721 ± 0.08
30 s	SPUR‐iG (Ours)	**6.6** ± **4.8**	**8.0** ± **5.9**	**0.816** ± **0.09**	**0.757** ± **0.012**
	Hybrid 2D/3D	7.3 ± 5.3	9.2 ± 6.6	0.773 ± 0.09	0.680 ± 0.022
	LLR	11.2 ± 7.3	11.1 ± 8.2	0.650 ± 0.02	0.541 ± 0.024

*Note:* Relative errors are computed within the brain (excluding CSF) and reported as mean ± standard deviation across voxels, averaged over all test subjects. SSIM is computed on axial brain slices and averaged across slices from all test subjects. Bold entries indicate values that are statistically significantly better than those of the other methods.

**FIGURE 3:**
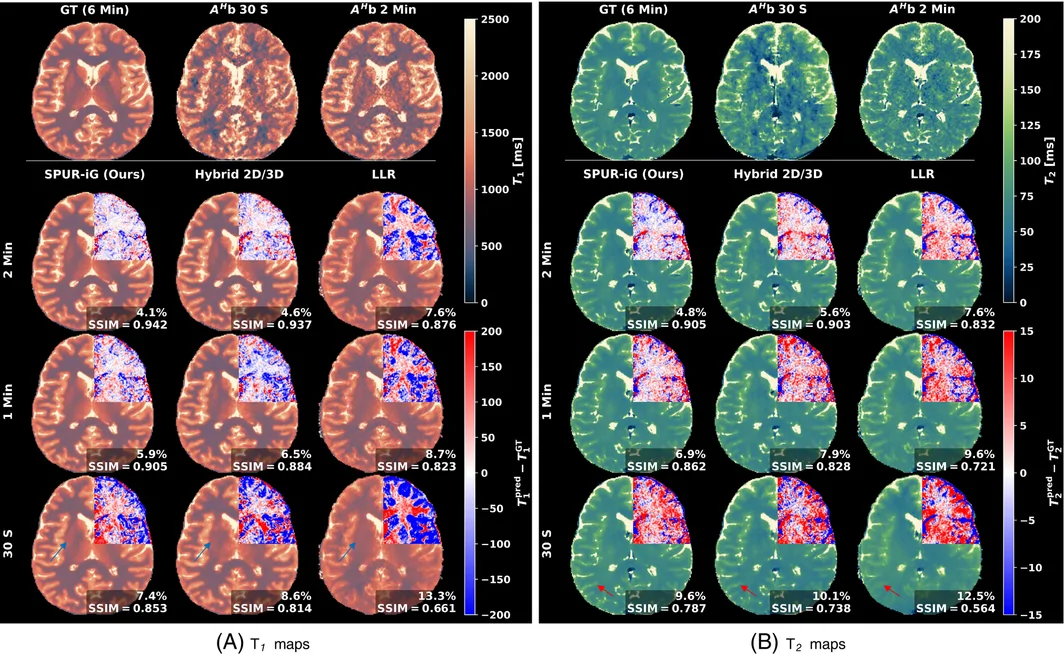
Qualitative examples of reconstructed $T_{1}$ (a) and $T_{2}$ (b) maps. The first row shows the 6 min reference (left) and maps from $A^{H}b$ for the 30 s and 2 min acquisitions (middle and right) as references. Subsequent rows present reconstructions at 2 min, 1 min, and 30 s, with columns comparing our method, the hybrid 2D/3D variant, and LLR. Error maps of the top‐right quadrant are overlaid on each image. Quantitative colormap (top) and error colormap (bottom) are shown to the right.

Representative qualitative results are presented in Figure 3. For 2 min scans, all methods produce reasonable maps, with ours showing the lowest residual errors. At 30 s, LLR yields overly smoothed maps that lose fine anatomical details. The hybrid method also exhibits some smoothing compared to our method, as highlighted by the arrows in Figure 3, with slightly lower SSIM values. SPUR‐iG better preserves fine structures while maintaining lower error overall.

### 3.3 Method Ablation

To assess the contribution of individual training stages, we compare the following variants:

1. *Full:* Our complete approach with all three training stages.
2. *GLEAM:* The model after the first two stages, before final fine‐tuning, with training time that matches *Full*.

To evaluate the effect of unroll iteration conditioning and weight sharing, we further compare:

3 *Full (uncond.):* Full training using an unconditioned model, that is, without unroll iteration conditioning.
4 *Full (uncond., no WS):* Same as *Full (uncond.)*, but without weight sharing in the GLEAM and fine‐tuning stages (resulting in a 6× increase in parameters).

**FIGURE 4:**
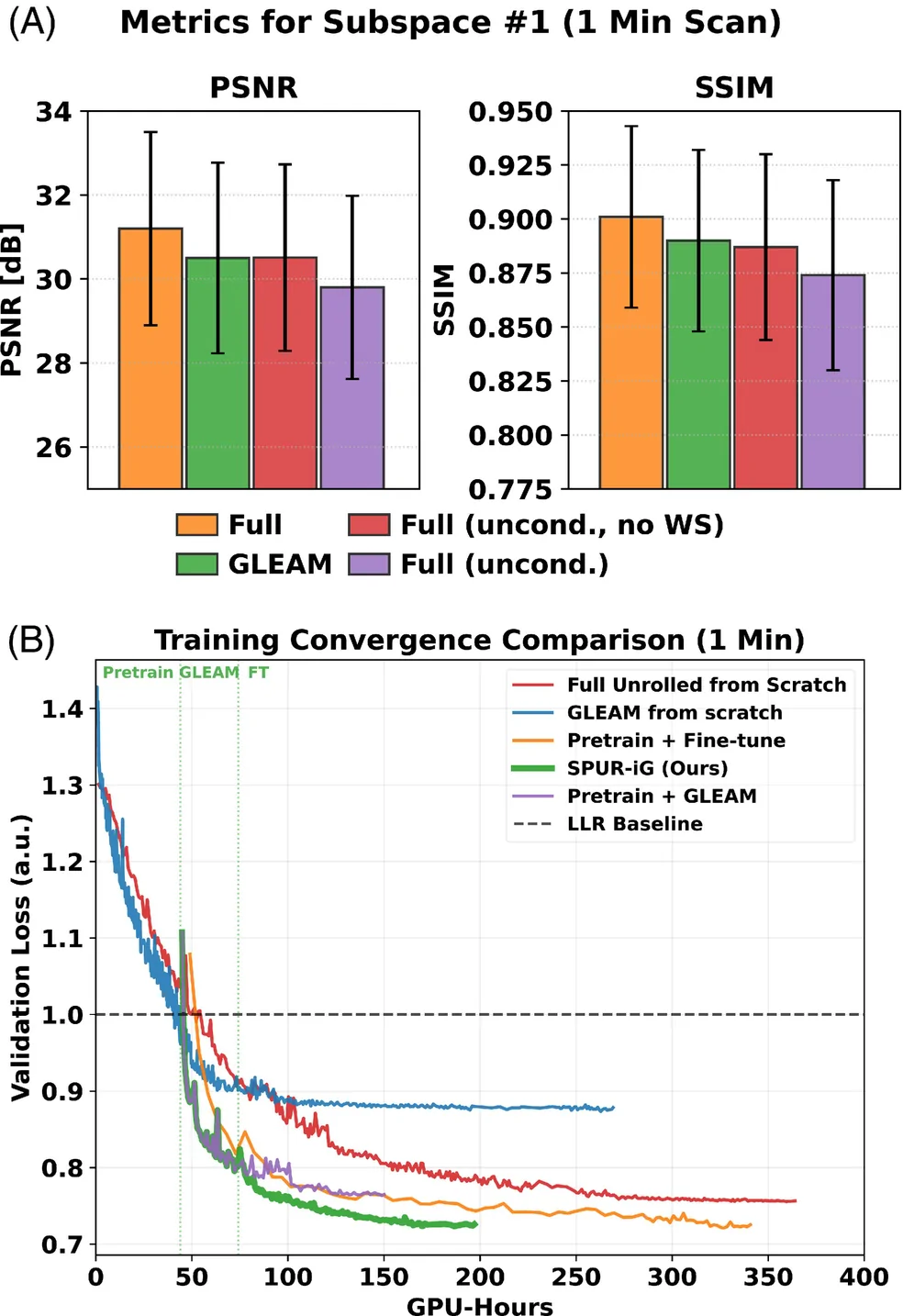
Training analysis for 1 min scans. (a) Ablation of training components: PSNR (left) and SSIM (right) for the first subspace coefficient map. Results are shown for the full approach (Full), training without final fine‐tuning (GLEAM), full training without unroll iteration conditioning (Full uncond.), and full training without conditioning or weight sharing (Full uncond., no WS). Similar trends are observed across other scan durations and subspace coefficient indices. (b) Training convergence comparison. Validation loss is plotted against GPU‐hours for five configurations: Full unrolled training from scratch (red), GLEAM from scratch (blue), Pretrain followed by fine‐tuning (orange), Pretrain followed by GLEAM (purple), and our full pipeline (green). Vertical dashed lines indicate the stage transitions for SPUR‐iG. The horizontal dashed line marks the LLR baseline loss. The validation set and loss computation are consistent across all runs. The pretraining stage uses a different set of inputs (e.g., LLR reconstructions at various scan durations), so its validation loss is not directly comparable; curves for methods that include pretraining begin at the GPU‐hours consumed by that stage. All configurations use the same iGROG‐based DC; with conventional NUFFT‐based DC, the per‐iteration cost, and therefore the training time of all methods, would be substantially higher.

Figure 4a shows PSNR and SSIM for the different training variants on 1 min scans (trends are similar for other durations). As can be seen, final fine‐tuning provides additional improvements on top of GLEAM. Additionally, iteration conditioning improves performance, and when conditioning is removed, weight sharing decreases performance.

To evaluate training efficiency, Figure 4b compares validation loss as a function of GPU‐hours for five training configurations. All configurations use iGROG‐based DC, so the reported GPU‐hours reflect this acceleration and isolate the effect of the staged training strategy. With conventional NUFFT‐based DC, the per‐iteration cost, and hence the training time of every configuration, would be substantially higher (4× to 15×, see Section 3.5). Full unrolled training from scratch converges slowly, as GC (required to fit in memory, as in our fine‐tuning stage) significantly increases the per‐iteration cost. GLEAM from scratch avoids this overhead and trains faster initially, but converges to a suboptimal point. Our staged pipeline addresses both limitations. Pretraining provides a strong initialization, and the GLEAM stage efficiently resolves the input distribution mismatch, together enabling rapid convergence to a good operating point. The subsequent full unrolled fine‐tuning further reduces the loss, confirming that end‐to‐end optimization is necessary to reach the best performance. Skipping the GLEAM stage (PT followed by fine‐tuning) eventually reaches a similar loss but converges more slowly. Overall, our full pipeline achieves the lowest loss in the fewest GPU‐hours.

**FIGURE 5:**
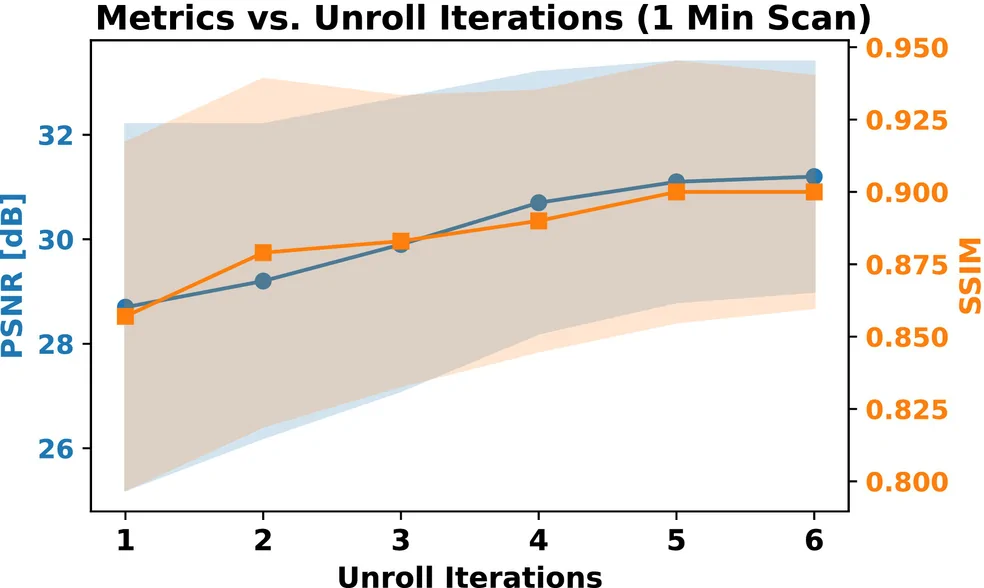
PSNR and SSIM for the first subspace coefficient map in 1 min scans, for models trained with different numbers of unroll iterations. Similar trends are observed across other scan durations and subspace coefficients.

Finally, Figure 5 reports PSNR and SSIM for models trained with a different number of unroll steps, showing that performance improves monotonically and converges around six unroll steps.

### 3.4 Out‐Of‐Distribution Generalization

**FIGURE 6:**
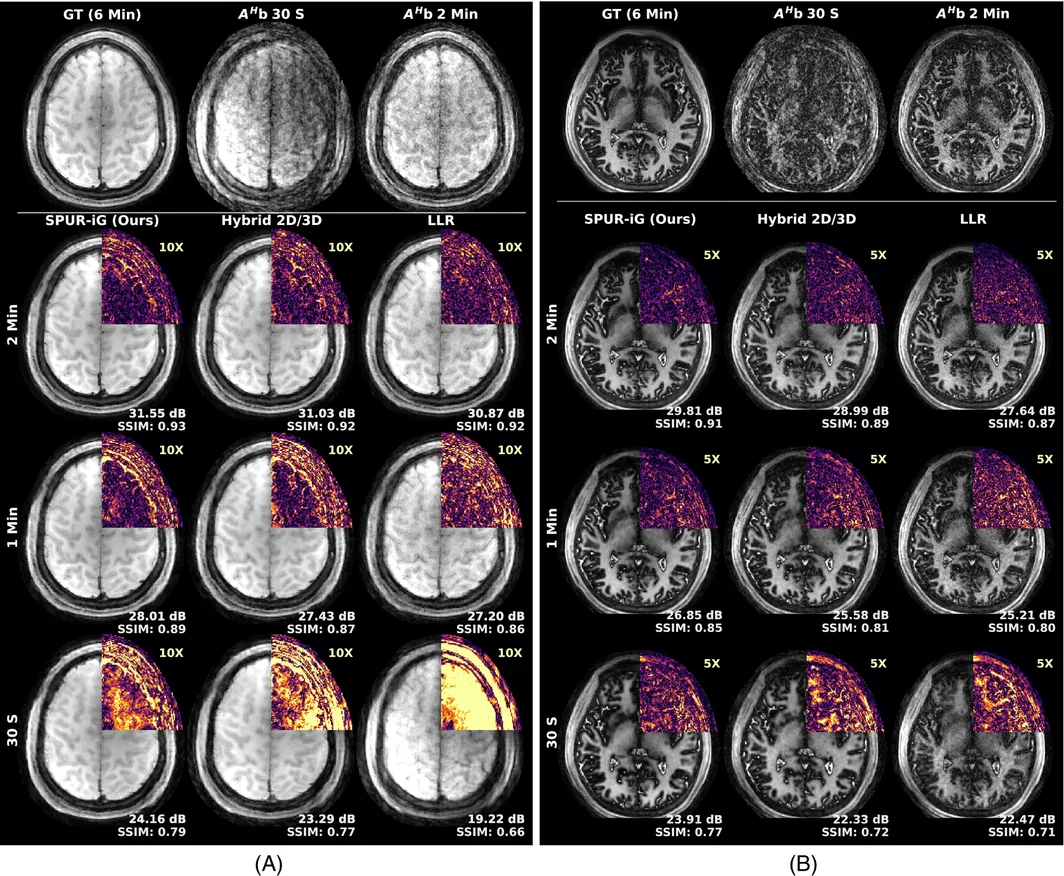
Reconstructions of the first balanced subspace coefficient (a) and the third unbalanced coefficient (b) for two slices from the same subject, scanned on an out‐of‐distribution scanner vendor. The layout follows Figure 2.

**FIGURE 7:**
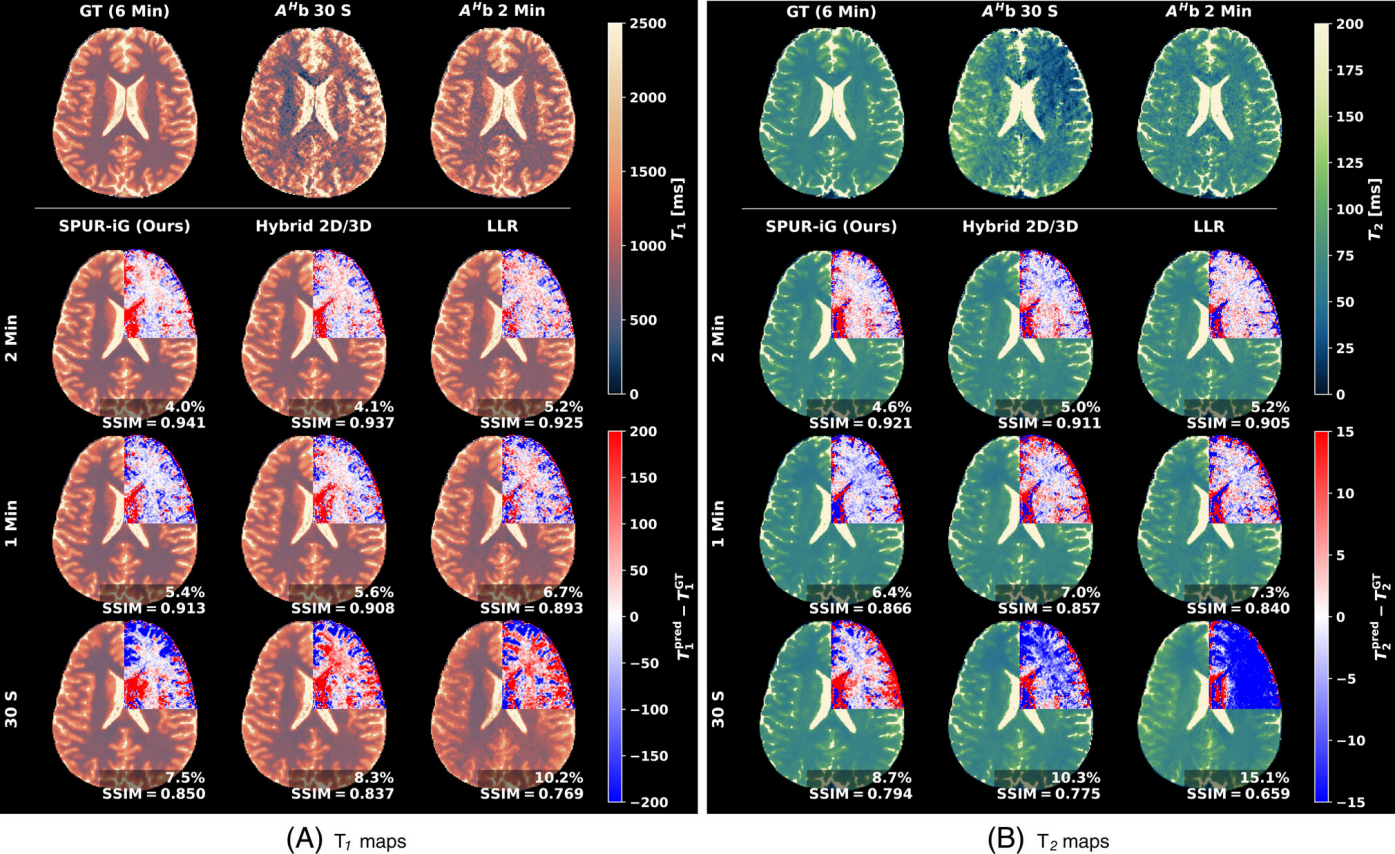
Qualitative examples of reconstructed $T_{1}$ (a) and $T_{2}$ (b) maps from an out‐of‐distribution vendor scan. The layout follows Figure 3.

To assess sensitivity to scanner vendor, we acquired data from a single subject on a Siemens 3 T system using the same sequence and reconstructed it using the exact same model and setup (which was trained exclusively on GE data). Figures 6 and 7 show the corresponding subspace reconstructions and quantitative maps, which exhibit trends similar to those observed on the in‐distribution test set.

### 3.5 Reconstruction Time

**FIGURE 8:**
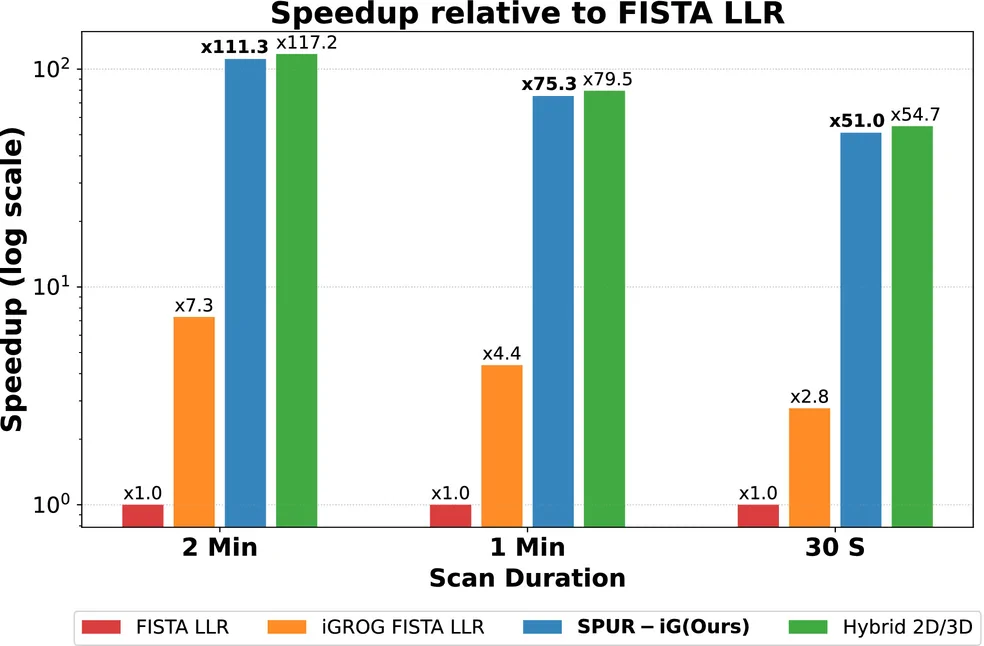
Relative reconstruction speedup compared to FISTA LLR with 40 iterations. The chart compares iGROG with FISTA LLR (40 iterations), our method with six unroll steps, and the hybrid 2D/3D unrolled variant with six unroll steps. For methods using iGROG, reconstruction times include gridding but exclude kernel training (which takes less than 25 s and can be performed in parallel to acquisition). For reference, the reconstruction times (in seconds) for each method and scan duration, from left to right, are: 1758.6, 241.7, 15.8, 15.0 (2 min); 986, 225.3, 13.1, 12.4 (1 min); and 601.7, 217.0, 11.8, 11.0 (30 s). All experiments are performed on a single A6000 GPU.

Figure 8 summarizes reconstruction speedups relative to FISTA LLR. All experiments were performed on a Linux workstation using a single NVIDIA A6000 GPU (48GB memory) with a PyTorch implementation. Our method achieves up to ×111 speedup for the 2 min acquisition and ×51 for the 30 s acquisition. Compared to iGROG FISTA LLR, the speedups are ×15 and ×18 for the 2 min and 30 s cases, respectively. Notably, reconstruction completes in less than 15 s for all acquisition durations. We assume iGROG INR training is performed during MRF acquisition after calibration data are obtained and therefore does not add to reconstruction time, while gridding is included in the reported reconstruction times. INR training operates on the calibration region of size $24^{3}\times 10$ and completes in under 25 s. Gridding is applied to the full acquired k‐space (with the largest case being the 2 min acquisition at 10(coils)×16(groups)×540(TRs)×1640(readout) k‐space samples) and completes in under 3 s; both use the same GPU. For reference, a single FFT‐based DC step after iGROG gridding takes less than 1.2 s across all scan durations, compared to 5–18 s for a comparable NUFFT‐based step (kernel size 4, oversampling factor 1.25). Speedup decreases for shorter scans as the DC cost scales with the size of the acquired k‐space. The hybrid 2D/3D variant is marginally faster than the 3D model (by less than 1 s), but both provide comparable computational efficiency.

## 4 Discussion

### 4.1 Quantitative Estimation

With aggressive undersampling, LLR requires stronger regularization to suppress noise, which introduces systematic bias and produces overly smooth maps (Figure 3, rightmost column). The patch‐based low‐rank constraint couples signal evolutions of neighboring voxels, and when a patch spans heterogeneous tissues, the resulting low‐rank approximation compresses the dynamic range of the estimated parameters: high values are pulled lower and low values are pushed higher. This is most evident at tissue boundaries, where the low‐rank assumption does not hold, but it also manifests more broadly within tissue regions. Although we jitter patch locations across iterations during LLR reconstruction to reduce blocking artifacts, the patch‐based constraint still couples signals from different tissue types, leading to biased values at boundaries (e.g., elevated tissue values for WM and reduced values for GM or CSF).

Our learned prior mitigates this effect by replacing patch‐based low‐rank constraints with a UNet denoiser. Unlike LLR, the UNet produces voxel‐wise predictions informed by convolutional receptive fields, allowing it to incorporate spatial context while preserving local tissue differences. In practice, this enables the network to recognize and respect boundaries rather than enforcing a shared representation across them. Residual averaging effects remain, especially at shorter scan durations, but they are substantially smaller. The 3D model further reduces this bias by leveraging volumetric context. Most of the remaining error appears in CSF, which is expected since this particular MRF sequence is primarily designed to optimize sensitivity for WM/GM [9].

Although our training objective is not explicitly designed to optimize tissue quantification, but rather to improve subspace coefficient reconstruction, Table 2 shows that our method improves quantitative performance, highlighting the benefit of the learned prior. Future work could integrate quantitative parameter estimation directly into the pipeline and jointly optimize for estimation accuracy, or optimize the learned prior for other downstream applications, such as multi‐compartment modeling.

In this work, acceleration is achieved by subsampling the number of spiral interleaves per group. An alternative and complementary approach is to reduce the number of TRs. However, for the FISP‐MRF sequence used here, the TR train is already relatively short, and further reduction would degrade $T_{2}$ sensitivity due to the loss of stimulated echoes at later TRs. For sequences with longer TR trains, jointly tuning both acceleration axes is an interesting direction for future work.

We use $B_{1}$ maps extracted from dummy group scans to correct $T_{2}$ bias when performing dictionary fitting. However, as scan duration decreases, the overhead from these groups becomes significant. Future work could integrate calibration‐free methods to estimate $B_{1}$ maps directly from reconstructed coefficient maps [51], reducing scan overhead and improving efficiency.

Finally, our training data consists of healthy subjects only. Validation on pathological cases (e.g., tumors, lesions, or other focal abnormalities) is outside the scope of this work and remains an important direction for future evaluation.

### 4.2 Coefficient Maps

Coefficient maps preserve richer temporal information than $T_{1}/T_{2}$ maps and are important for emerging downstream applications such as multi‐compartment modeling and clinical contrast synthesis. While $T_{1}$ and $T_{2}$ maps are the primary output for many applications, evaluating the coefficient maps directly is also important, as the dictionary fitting step provides inherent robustness that can mask residual reconstruction artifacts, which, although not visible in the quantitative maps, may impact these other downstream applications. As shown in Table 1, our method consistently outperforms the baselines in PSNR and SSIM, with performance gaps widening as scan duration decreases. The improvements come from two factors: the learned prior, which captures more accurate image statistics than hand‐crafted regularization, and the 3D UNet architecture, which leverages volumetric correlations across slices for stronger regularization. The proposed training strategy enables training and deployment of such a model, supporting a more complex and accurate representation of image statistics and, consequently, improved reconstructions.

The MRF scans used in this work utilize an optimized spatio‐temporal k‐space sampling strategy based on the TGAS approach [9]. Future work could further improve performance by refining sampling trajectories within the reconstruction training process [23], which may be particularly valuable for very short acquisitions such as 30 s.

### 4.3 Training Strategy

Figure 4a,b evaluate the impact of different components of our framework:

1. Pretraining is a key element of our approach. By avoiding unrolling and DC, it enables efficient training with extensive data augmentation, which improves generalization and robustness. Pretraining also substantially accelerates convergence: GLEAM from scratch plateaus at a suboptimal loss, and full unrolled training from scratch converges much more slowly, while our pipeline starting from pretraining reaches a lower loss in substantially fewer GPU‐hours.
2. Fine‐tuning with full unrolling provides a consistent improvement by directly optimizing the final reconstruction loss.
3. Conditioning on the unroll iteration index improves performance over unconditional variants. It simplifies the denoiser's task by explicitly providing the expected artifact and noise‐level distribution, rather than requiring the model to infer these solely from the input. In unconditional training, not sharing weights performs better than sharing, since the model no longer has to infer denoising strength from the input. However, both remain inferior to the conditioned version, which amortizes learning across noise regimes, effectively enlarging the dataset. The cost of conditioning is negligible, adding less than 1% to the model parameters.

The staged pretraining pipeline is motivated primarily by training efficiency. Figure 4b shows that our pipeline substantially reduces wall‐clock training time compared to full unrolled training from scratch. The key insight is that much of the unrolled training time is spent learning effective denoising capabilities, which can be done outside of the slow unrolled framework; this is precisely what our pretraining stage accomplishes. GLEAM is then used to quickly align the denoiser's input distribution with the intermediate outputs encountered during unrolled reconstruction, reaching a good starting point for full unrolled fine‐tuning. This not only accelerates training but also reaches a lower final loss than training from scratch.

Although the training set consists exclusively of data acquired on a 3 T GE system, Figures 6 and 7 demonstrate that the method generalizes to an out‐of‐distribution vendor without retraining or fine‐tuning, indicating cross‐vendor robustness. This is despite hardware differences including the coil array, nominal field strength, and ADC dwell time. This degree of robustness is consistent with findings from other unrolled reconstruction approaches; however, further evaluation across additional scanners and sites is warranted, and performance degradation could likely be mitigated by incorporating a small amount of out‐of‐distribution data during training or fine‐tuning.

Finally, while demonstrated here for MRF, the proposed 3D unrolled training strategy is generally applicable to other large‐scale imaging problems. Applications such as high‐resolution structural and dynamic 3D imaging [52] may particularly benefit from its scalability and efficiency.

## 5 Conclusions

We propose a fully 3D unrolled MRF reconstruction framework with a staged training strategy that makes large‐scale training more efficient within practical compute limits. By combining efficient iGROG‐based DC with fast 3D UNet‐based inference, the method achieves rapid reconstruction while consistently improving quality, supporting acquisitions as short as 30 s and preserving fidelity in both subspace coefficients and quantitative maps.

This represents an important step toward enabling short clinical acquisitions with fast reconstructions that can directly produce quantitative maps or synthesize clinical contrasts for routine use. Future work may explore alternative training objectives tailored to downstream applications, such as directly optimizing $T_{1}$/$T_{2}$ estimation or multi‐compartment fitting. Another interesting direction is to extend the staged training strategy to even higher‐resolution MRF such as 360μm isotropic [47], while keeping computation and memory demands tractable.

## Funding

This was supported by National Institutes of Health (R01 EB033206, R01 MH116173, R01 EB019437).

## Supporting information


**Section S1:** Implementation details.
**Section S1.1:** Model.
**Section S1.2:** Pretraining.
**Section S1.3:** GLEAM stage.
**Section S1.4:** Fine‐tuning.
**Section S1.5:** LLR reconstruction.
**Section S2:** Subspace energy equalization.

## Data Availability

Research data are not shared.
