# Supplementary material for: Fully 3D Unrolled Magnetic Resonance Fingerprinting Reconstruction via Staged Pretraining and Implicit Gridding
Source: Magn Reson Med. 2026 Jul 29;96(5):2516–29. doi: 10.1002/mrm.70500 (PMC13527266; doi:10.1002/mrm.70500)
Supplement: Supplementary file 1 [file MRM-96-2516-s001.pdf]

S1 | IMPLEMENTATION DETAILS

S1.1 | Model

We implemented both 3D and 2D U-Nets with four encoder–decoder levels. Each level contained a residual block consisting of two convolutional layers with kernel size 3, followed by group normalization [49] (8 groups) and SiLU activations [50]. Additionally, a Squeeze-and-Excitation block [51] with reduction factor 16 was applied after each residual block.

Inputs are $k = 5$ complex subspace volumes, split into $C = 10$ real/imaginary channels and whitened jointly for zero mean and unit covariance.

Unroll step conditioning was implemented by mapping the unroll index i to sinusoidal embeddings with dimension 48, followed by an MLP generating scale and shift parameters (γ_i, β_i), injected into each block via FiLM-style modulation [38] $h' = (1 + \gamma_i) \odot h + \beta_i$.

Unrolled reconstruction is initialized with $\mathcal{A}^H \mathbf{b} \cdot s$, where $s = \|\mathbf{b}\|_2 / \|\mathcal{A}\mathcal{A}^H \mathbf{b}\|_2$ is a scaling factor that aligns the norms of the initialization and the acquired k-space. We found this normalization reduces the number of required unrolled steps.

Training used Adam (initial learning rate 5×10^{-3}, halved after 20 epochs without validation improvement). Pretraining required 48 GPU hours, GLEAM training 27 GPU hours, and fine-tuning 100 GPU hours, all on a single NVIDIA A6000 (48 GB).

The pretraining stage uses approximately 75% of GPU memory, as it does not involve DC updates. The GLEAM and fine-tuning stages both saturate the full 48 GB of the A6000 GPU. In both cases, the peak memory during backpropagation is approximately that of a single unroll step: GLEAM achieves this by detaching the computation graph after each step, while full unrolled training uses gradient checkpointing to achieve the same effective footprint.

S1.2 | Pretraining

In pretraining, input types were heuristically matched with iteration indices as

$$\begin{aligned} \mathcal{A}^H \mathbf{b} &\mapsto 1, \\ \boldsymbol{\alpha}_{\text{LLR}}^{\text{30s}} &\mapsto 2, \\ \boldsymbol{\alpha}_{\text{LLR}}^{\text{1min}} &\mapsto 2, \\ \boldsymbol{\alpha}_{\text{LLR}}^{\text{2min}} &\mapsto 3, \\ \boldsymbol{\alpha}_{\text{LLR}}^{\text{6min}} &\mapsto k, \quad k \sim \mathcal{U}[4,6]. \end{aligned}$$

Here, the highest-quality input $\boldsymbol{\alpha}_{\text{LLR}}^{\text{6min}}$, which also serves as the ground truth, is assigned a randomly sampled iteration index k drawn uniformly from $\mathcal{U}[4,6]$. Data augmentations included random rotations ($\leq 15°$), translations (up to ± 5 voxels), and scaling in $[0.98, 1.02]$.

Tissue Aware Loss

We define a tissue-aware spatially weighted L_1 which we use during pretraining:

$$\mathcal{L}_{\text{spatial}}(\hat{\boldsymbol{\alpha}}, \boldsymbol{\alpha}) = \sum_{v \in \{\text{BG,CSF,Tissue}\}} \lambda_v \, \|\hat{\boldsymbol{\alpha}}_v - \boldsymbol{\alpha}_v\|_1, \tag{S1}$$

where $\boldsymbol{\alpha}_v$ and $\hat{\boldsymbol{\alpha}}_v$ denote the ground truth and estimated coefficient images restricted to region v, obtained from segmentation masks computed from the GT reconstruction. Here, BG denotes background voxels and Tissue comprises all remaining non-CSF voxels (including WM, GM, and any other tissue). We set $\lambda_{\text{Tissue}} = 10$ and $\lambda_{\text{BG}} = \lambda_{\text{CSF}} = 1$. Background is downweighted because it dominates the volume; CSF is downweighted because physiological pulsation, flow, and CSF motion induce spin-history variations that cause signal differences across acquisition groups, making quantitative estimates in CSF intrinsically less reliable than in relatively static tissues such as WM and GM [52]. As these variations are physiological and cannot be eliminated, we downweight these regions to avoid fitting non-deterministic signal differences.

S1.3 | GLEAM stage

In the GLEAM stage, supervision is applied to all intermediate unroll outputs $\{\boldsymbol{\alpha}_k\}_{k=1}^K$. Additionally, we augment the L_1 loss with a multi-scale structural similarity (MS-SSIM). The loss is defined as

$$\mathcal{L}_{\text{GLEAM}} = \sum_{k=1}^{K} \mathcal{L}_{\text{spatial}}(\boldsymbol{\alpha}_k, \boldsymbol{\alpha}_{\text{gt}}) + \lambda_{\text{SSIM}}\, \mathcal{L}_{\text{MS-SSIM}}(\boldsymbol{\alpha}_K, \boldsymbol{\alpha}_{\text{gt}}),$$

where $\boldsymbol{\alpha}_{\text{gt}}$ denotes the ground truth. We apply early stopping when the validation loss has not decreased for 10 epochs, without learning rate reduction, to prevent the model from converging to a local minimum that is difficult to escape during subsequent fine-tuning. Due to memory constraints, MS-SSIM is computed on 30 randomly selected axial slices per volume. We use full volumes as input with a batch size of 1, and employed gradient accumulation with a factor of 4.

S1.4 | Fine-tuning

The fine-tuning stage uses the same loss, batch size and gradient accumulation formulation as GLEAM, but supervision is applied only to the final output.

S1.5 | LLR Reconstruction

Reconstruction used patches of size $10 \times 10 \times 10$ voxels with random shifts per iteration, and SVD thresholds of 5×10^{-5} (6 min), 1×10^{-4} (2 min, 1 min), and 1.5×10^{-4} (30 s).

S2 | SUBSPACE ENERGY EQUALIZATION

Learned denoisers benefit from having roughly uniform energy across input channels, as this stabilizes training and is consistent with the standard practice of normalizing network inputs. In the subspace reconstruction setting, the time series is represented by Eq. (1), where the standard basis $\boldsymbol{\Phi}$ is obtained by computing the SVD of the MRF dictionary, which results in order-of-magnitude variations in the energy of each subspace image. However, we can perform a change of basis using a unitary matrix $\mathbf{Q} \in \mathbb{C}^{k \times k}$,

$$\mathbf{x} \approx \boldsymbol{\Phi\alpha} = \boldsymbol{\Phi}\mathbf{Q}^H\mathbf{Q}\boldsymbol{\alpha} = \tilde{\boldsymbol{\Phi}}\tilde{\boldsymbol{\alpha}}, \tag{S2}$$

where under the new basis $\tilde{\boldsymbol{\Phi}} = \boldsymbol{\Phi}\mathbf{Q}^H$, the subspace images $\tilde{\boldsymbol{\alpha}} = \mathbf{Q}\boldsymbol{\alpha}$ have a more uniform energy distribution across the k channels.

Given $\boldsymbol{\alpha} \in \mathbb{C}^{k \times N_v}$, we define the channel energy matrix

$$\mathbf{E} = \boldsymbol{\alpha}\boldsymbol{\alpha}^H \ \in \ \mathbb{C}^{k \times k}, \tag{S3}$$

so that $\mathbf{E}_{ii}$ is the energy of the i-th channel. We seek a unitary matrix $\mathbf{Q}$ such that the transformed images $\tilde{\boldsymbol{\alpha}} = \mathbf{Q}\boldsymbol{\alpha}$ have equal energy in every channel, i.e. $\text{diag}(\mathbf{QEQ}^H)$ is constant. Since $\mathbf{Q}$ is unitary, this is merely a change of basis and preserves the reconstruction solution. First, we diagonalize the energy matrix as

$$\mathbf{E} = \mathbf{U}\boldsymbol{\Lambda}\mathbf{U}^H, \tag{S4}$$

where $\mathbf{U}$ is unitary and $\boldsymbol{\Lambda} = \text{diag}(\lambda_1, \dots, \lambda_k)$. Now let $\mathbf{F} \in \mathbb{C}^{k \times k}$ be any unitary matrix whose entries all have equal magnitude, $|F_{ij}| = 1/\sqrt{k}$ (for example, the normalized DFT matrix). Setting

$$\mathbf{Q} = \mathbf{F}\mathbf{U}^H, \tag{S5}$$

we obtain

$$\mathbf{QEQ}^H = \mathbf{F}\mathbf{U}^H\big(\mathbf{U}\boldsymbol{\Lambda}\mathbf{U}^H\big)\mathbf{U}\mathbf{F}^H = \mathbf{F}\boldsymbol{\Lambda}\mathbf{F}^H. \tag{S6}$$

The i-th diagonal entry of (S6) is

$$\big[\mathbf{F}\,\boldsymbol{\Lambda}\,\mathbf{F}^H\big]_{ii} \;=\; \sum_{j=1}^{k} |F_{ij}|^2\,\lambda_j \;=\; \frac{1}{k}\sum_{j=1}^{k}\lambda_j \;=\; \frac{\text{tr}(\mathbf{E})}{k}, \tag{S7}$$

so every channel of $\tilde{\boldsymbol{\alpha}} = \mathbf{Q}\boldsymbol{\alpha}$ has exactly the same energy.

In practice, to find a $\mathbf{Q}$ that generalizes well across the dataset, we estimate the energy matrix by averaging over all N samples in the validation set:

$$\bar{\mathbf{E}} = \frac{1}{N}\sum_{n=1}^{N} \boldsymbol{\alpha}_n \boldsymbol{\alpha}_n^H \tag{S8}$$

and apply the above procedure, which results in approximately equal energy distribution across channels.
